# Comparative Evaluation of Oxidative Stress, Cortisol, Inflammatory Response, and Systemic CO_2_ Dynamics in Dogs Undergoing Laparoscopic and Open Ovariectomy

**DOI:** 10.3390/antiox15070886

**Published:** 2026-07-17

**Authors:** Sandra Čechovičienė, Ieva Šidlauskaitė, Birutė Karvelienė, Ieva Sarapinienė, Ieva Čiapienė, Vita Riškevičienė, Dalia Juodžentė

**Affiliations:** 1 L. Kriaučeliūnas Small Animal Clinic, Faculty of Veterinary, Veterinary Academy, Lithuanian University of Health Sciences, Tilžės Str. 18, 47181 Kaunas, Lithuaniadalia.juodzente@lsmu.lt (D.J.); 2 Institute of Cardiology, Lithuanian University of Health Sciences, Sukilėlių Ave 15, 50162 Kaunas, Lithuania; 3 Department of Veterinary Pathobiology, Faculty of Veterinary, Veterinary Academy, Lithuanian University of Health Sciences, Tilžės Str. 18, 47181 Kaunas, Lithuania

**Keywords:** cortisol, cytokines, dog, laparoscopic, laparotomy, oophorectomy, pneumoperitoneum, surgical stress response

## Abstract

A key distinction between open and laparoscopic surgery is the use of CO_2_ insufflation to create pneumoperitoneum and establish the operative field. This study compared the effects of laparoscopic and open ovariectomy (OVE) on plasma cortisol, oxidative stress (OS), systemic inflammation, and systemic CO_2_ dynamics in healthy dogs anesthetized under a standardized protocol. Thirty-eight healthy female dogs were enrolled in the study and allocated to the laparotomy (LPTOVE, *n* = 19) or laparoscopic (LAPOVE, *n* = 19) OVE group. OS was assessed using total oxidant status (TOS), total antioxidant status (TAS), and oxidative stress index (OSI). Systemic inflammation was evaluated by TNF-α and IL-6 concentrations, whereas systemic CO_2_ dynamics were assessed using plasma total CO_2_ (TCO_2_) and end-tidal CO_2_ (EtCO_2_). Blood samples were collected prior to sedation (T0), prior to surgical incision (T1), prior to surgical closure in the LPTOVE group or CO_2_ desufflation in the LAPOVE group (T2), and 2 h postoperatively (T3). Cortisol concentrations at T2 and T3 were significantly higher in the LPTOVE group than in the LAPOVE group. No significant intergroup differences were observed in TOS, TAS, OSI, or TCO_2_. In the LPTOVE group, TOS significantly decreased at T3, whereas in the LAPOVE group, TOS mildly increased at T1 and OSI significantly decreased at T2. No measurable TNF-α or IL-6 concentrations were detected at the evaluated time points. EtCO_2_ levels were higher in the LAPOVE group at the end of surgery. These findings suggest that laparoscopic OVE was associated with lower perioperative cortisol concentrations, while systemic redox status, TNF-α and IL-6 concentrations, and carbon dioxide-related parameters remained largely comparable between surgical approaches.

## 1. Introduction

Ovariectomy is among the most performed surgical procedures in dogs and is primarily undertaken for population control, disease prevention, and behavioral management [1,2,3]. Over recent decades, minimally invasive techniques, particularly laparoscopy, have been increasingly adopted in veterinary medicine, improving outcomes of procedures such as ovariohysterectomy (OHE) and ovariectomy (OVE) [4,5,6,7,8,9].

The key distinction between open and laparoscopic surgery lies in the creation of pneumoperitoneum (PNP) through CO_2_ insufflation, which is essential for establishing an adequate operative field. Most studies have focused on the cardiovascular and respiratory effects of PNP, where increased intra-abdominal pressure (IAP) causes cranial diaphragm displacement, leading to reduced venous return, increased vascular resistance, decreased cardiac output, and impaired lung function [10,11,12,13,14]. In contrast, the impact of CO_2_ insufflation and its peritoneal absorption on the hemodynamic system has not been fully elucidated. Fukushima et al. reported that a 30 min PNP in dogs resulted in mild hypercapnia, respiratory acidosis, and a compensatory increase in blood bicarbonate levels [15]. In our previous study, an increase in end-tidal CO_2_ (EtCO_2_) was observed at the end of laparoscopic OVE performed under IAP of 10–12 mmHg. This elevation may be associated with CO_2_ absorption during PNP, potentially leading to hypercapnia [16]. However, further studies are required to clarify the underlying mechanisms of this observation.

Elevated IAP has also been reported to contribute to increased oxidative stress (OS) [12,13,17]. A previous study demonstrated that increasing IAP to 15 mmHg for 60 min was associated with a reduction in total antioxidant status (TAS) and an elevation in oxidative stress index (OSI) at 90 min and 24 h after deflation of CO_2_ PNP. In contrast, no significant changes were observed at an IAP of 12 mmHg [17]. Reactive oxygen species (ROS) are continuously generated during normal aerobic metabolism and participate in multiple physiological functions, including intracellular signaling and immune regulation [18]. Under physiological conditions, oxidant production is balanced by enzymatic and non-enzymatic antioxidant defense mechanisms. Disturbance of this balance in favor of oxidants results in oxidative stress, which may damage lipids, proteins, and nucleic acids [18,19,20,21,22]. Under conditions of excessive oxidant production, wound healing is delayed due to cytotoxic effects on dermal fibroblasts and keratinocytes, enhanced inflammatory cell chemotaxis, and disruption of antioxidant defense systems [22,23,24].

In veterinary medicine, surgical trauma is known to induce ROS production that may overwhelm antioxidant defenses and result in measurable OS in dogs. Accordingly, OS has been widely used as a biomarker to evaluate the effects of different surgical techniques [11,25,26,27,28,29,30,31]. Previous studies have generally shown that laparoscopic OVE results in lower oxidative stress than open OVE [11,26,27,29,31]. Similarly, our previous study indicated reduced intraoperative OS during laparoscopic OVE compared with open OVE. However, the observed postoperative reduction in TAS and increase in OSI at 2 h suggest comparable postoperative tissue stress between techniques [16], indicating that OS alone may not fully reflect the complexity of the surgical stress response.

In addition to OS, plasma cortisol levels are widely used to assess surgical stress and pain in both human and veterinary medicine [32,33,34,35]. Studies investigating plasma cortisol responses to laparoscopic and open OHE or OVE have primarily focused on the postoperative period, where cortisol concentrations are typically elevated after open procedures [32,33,36,37]. Moreover, a previous study demonstrated that cortisol levels were significantly increased 40 min after PNP relative to baseline across four anesthetic groups [34]. However, data on perioperative cortisol dynamics, particularly in relation to oxidative stress responses, remain limited and require further investigation.

Surgical and anesthetic trauma induce a specific inflammatory response characterized by complex pathophysiological processes and activation of cytokine-mediated cascades [38,39], which are closely associated with increased OS [40,41]. This response plays a critical role in initiating tissue repair. However, when prolonged or dysregulated, it may lead to adverse outcomes such as persistent pain and immunosuppression [42,43]. Among the cytokines released following tissue injury, interleukin-6 (IL-6) is considered one of the most informative indicators of the magnitude of surgical trauma and the ensuing inflammatory response [31,44,45]. Despite its relevance, only one study has evaluated IL-6 dynamics in dogs undergoing laparoscopic or open OVE, reporting a transient increase 2 h after laparoscopic OVE with a return to baseline by day 7 [31]. Tumor necrosis factor alpha (TNF-α) is another pro-inflammatory cytokine with potential as a biomarker of surgical trauma in dogs, although evidence remains limited. One study demonstrated its involvement in the early upregulation of inflammatory processes following injury, with higher concentrations detected in wound fluid than in serum, whereas another reported only low TNF-α activity with no significant differences compared to controls [46,47].

A review of the available literature indicates that surgical stress during laparoscopic and open OVE cannot be adequately characterized by a single biomarker, and available data on the combined assessment of CO_2_ pneumoperitoneum-induced alterations, oxidative stress, endocrine response, and inflammation remain limited. Building on our previous study evaluating plasma cortisol and oxidative stress responses in laparoscopic and open OVE [16], the present study extends this approach by increasing the sample size, incorporating pro-inflammatory cytokines (IL-6 and TNF-α) as additional markers of systemic stress, and assessing total CO_2_ levels in blood to further elucidate the effects of CO_2_ PNP on systemic CO_2_ dynamics.

The aim of this study was to evaluate the effects of laparoscopic and open ovariectomy on plasma cortisol levels, oxidative stress, the systemic inflammatory response, and systemic CO_2_ dynamics in healthy dogs under a standardized anesthesia protocol.

## 2. Materials and Methods

### 2.1. Animals

This clinical study was conducted in the Dr. L. Kriauceliunas Small Animal Clinic, Veterinary Faculty, Lithuanian University of Health Sciences. The animal study protocol was approved by the Ethics Committee of the Lithuanian University of Health Sciences (protocol code Nr. 2024-BEC3-T-009, approved on 12 March 2024). Written informed consent was obtained from all dog owners prior to their inclusion in the study.

A total of 38 client-owned, clinically healthy female dogs scheduled for elective neutering were included in this study. For inclusion, all dogs were required to be in the anestrus phase of the estrous cycle and classified as American Society of Anesthesiologists (ASA) physical status I, based on anamnesis, physical examination, complete blood count, and serum biochemistry analysis.

The animals were allocated into two groups according to the owners’ choice of surgical technique: the laparotomy ovariectomy group (LPTOVE, *n* = 19) and laparoscopic ovariectomy group (LAPOVE, *n* = 19). Dogs in the LPTOVE group had a mean age of 36.37 ± 5.58 months and a mean body weight of 13.36 ± 1.72 kg. This group consisted of 18 mixed-breed dogs and one Cavalier King Charles Spaniel. Dogs in the LAPOVE group had a mean age of 22.42 ± 3.14 months and a mean body weight of 14.35 ± 2.25 kg. This group included 12 mixed-breed dogs and 7 purebred dogs: one Dachshund, one Standard Poodle, one Border Collie, one Curly Coated Retriever, one Shiba Inu, one Dobermann, one Jack Russell Terrier, and one Lagotto Romagnolo.

### 2.2. Anesthesia and Analgesia

All dogs were anesthetized using a standardized general anesthesia protocol following a 12 h fasting period. For sedation, all animals received methadone (0.5 mg kg^−1^; Richter Pharma AG, Wels, Austria) and dexmedetomidine (3 µg kg^−1^; Orion Corporation, Espoo, Finland) administered intramuscularly. After administration of sedatives, the dogs were kept in a quiet room for 15 min under the direct supervision of an anesthesiologist. An intravenous catheter was placed in the cephalic vein 15 min after sedation. Anesthesia was induced with propofol (1–2 mg kg^−1^; Accord Healthcare B.V., Utrecht, The Netherlands) to effect, until loss of palpebral and gag reflexes. Following endotracheal intubation with an appropriately sized tube, anesthesia was maintained with sevoflurane (SVO) (vaporizer 2.0%; AbbVie S.r.l., Campoverde di Aprilia (LT), Italy) in 100% oxygen (1.00 L min^−1^). A 0.9% sodium chloride solution (Braun Melsungen AG, Melsungen, Germany) was administered intravenously at a rate of 5 mL kg^−1^ per hour using an infusion pump (Braun, Melsungen, Germany), starting 15 min after sedation and continuing until complete recovery from anesthesia. In the LAPOVE group, animals were mechanically ventilated using a volume-controlled ventilation mode following the induction of PNP and maintained until CO_2_ deflation. The tidal volume (Vt) was set at 10 mL kg^−1^. The inspiratory-to-expiratory (I:E) ratio was set at 1:3 for dogs weighing < 10 kg and at 1:2 for dogs weighing ≥ 10 kg. The airway pressure limit was set to 20 cmH_2_O. Anesthesia time was defined as the period from sedation to discontinuation of SVO administration. At the end of anesthesia, atipamezole (30 µg kg^−1^; Eurovet Animal Health B.V., Bladel, The Netherlands) was administered subcutaneously as a dexmedetomidine antagonist. Postoperative analgesia included buprenorphine (0.015 mg kg^−1^; Richter Pharma AG, Wels, Austria) administered intramuscularly and meloxicam (0.2 mg kg^−1^; Chanelle Pharmaceuticals Manufacturing Ltd., Loughrea, Co. Galway, Ireland) administered subcutaneously after discontinuation of SVO [16].

The depth of anesthesia and physiological parameters were monitored by an anesthesiologist and recorded at 5 min intervals throughout the procedure, with baseline measurements obtained 15 min after administration of methadone and dexmedetomidine, and monitoring continued until the end of anesthesia. The following variables were assessed: heart rate (HR; beats min^−1^), respiratory rate (RR; breaths min^−1^), systolic arterial pressure (SAP; mmHg), diastolic arterial pressure (DAP; mmHg), oxygen saturation (SpO_2_; %), and end-tidal carbon dioxide (EtCO_2_; mmHg). These parameters were measured using a veterinary patient monitoring system (iM8 VET, Edan, Germany). Body temperature (BT; °C) was measured using an electronic thermometer. SAP and DAP were determined using a non-invasive oscillometric method. Oxygen saturation was measured by pulse oximetry (OXY-100 VET, Gima, Fara d’Adda, Italy), and EtCO_2_ was measured using a capnograph integrated into the anesthesia machine (WATO EX-35, Mindray, Shenzhen, China). An electronic circulating heating pad (Shenzhen Anpan Health Industry Co., Ltd., Shenzhen, China) was used to maintain BT within the range of 38.0–39.3 °C [16].

### 2.3. Surgical Procedure

Animals were placed in dorsal recumbency. Trichotomy of the surgical site and aseptic preparation of the abdominal skin were performed in both groups. Laparotomy OVE and laparoscopic OVE were performed by the same veterinary surgical team. Surgical time was defined as the interval from skin incision to completion of skin closure [16].

For the LPTOVE group, ovariectomy was performed via laparotomy. A ventral midline celiotomy was made caudal to the umbilicus, with incision length determined by the surgeon according to the size of the animal and extended intraoperatively as needed to allow ligation of the suspensory ligaments and ovarian pedicles with minimal traction. The skin was incised using a No. 11 scalpel blade (AMBRO, Naples, Italy), and the subcutaneous tissues were bluntly and sharply dissected along the midline and separated from the abdominal fascia with Mayo scissors (KLS Martin SE & Co. KG., Tuttlingen, Germany). Hemostasis was achieved using bipolar electrosurgical equipment (KLS Martin SE & Co. KG., Tuttlingen, Germany). The left ovary was identified following localization of the left uterine horn using a spay hook (KLS Martin SE & Co. KG., Tuttlingen, Germany), then grasped at the level of the suspensory ligament with hemostatic forceps (KLS Martin SE & Co. KG., Tuttlingen, Germany) and dissected from surrounding tissues using a bipolar vessel-sealing device (KLS Martin SE & Co. KG., Tuttlingen, Germany). The same procedure was repeated for the right ovary. After confirming the absence of hemorrhage, the abdominal incision was routinely closed: the abdominal fascia and subcutaneous tissues were sutured in a simple continuous pattern, and the skin was closed intradermally using synthetic absorbable monofilament polydioxanone suture material (Surgicryl^®^, SMI AG, Saint-Vith, Belgium) [16].

The LAPOVE surgeries were performed using a 2-portal laparoscopic technique. The video monitor was positioned at the cranial end of the patient. In each dog, a 10 mm trocar-cannula assembly (VersaOne™, COVIDIEN™, North Haven, CT, USA) was inserted 1 cm caudal to the umbilicus on the ventral midline using a modified Hasson technique [48]. Pneumoperitoneum (PNP) was established with CO_2_ insufflation using an automatic insufflator (DF-32O Automatic Insufflator, Micon Medizintechnik GmbH, Kaltenkirchen, Germany), and intra-abdominal pressure was maintained at 10–12 mmHg throughout the procedure. A 5 mm, 30°, 30 cm laparoscope (Dr. Fritz, Dr. Fritz Endoscopes GmbH, Buchheim, Germany) was inserted through the primary cannula to allow inspection of the abdominal cavity. A second 10 mm trocar was then placed paramedial to the first trocar, and the laparoscope was repositioned from the caudal to the cranial cannula [4]. Each ovary was grasped at the level of the ovarian bursa using laparoscopic grasping forceps and temporarily suspended to the abdominal wall and suspensory ligament with 1-0 polydioxanone suture. The ovaries were subsequently dissected from surrounding tissues using a bipolar electrosurgical vessel-sealing device (KLS Martin SE & Co. KG., Tuttlingen, Germany) and removed through the caudal cannula. After desufflation and removal of the cannulas, the abdominal incisions were closed using the same suturing technique as described for the LPTOVE group [16].

### 2.4. Analysis of Blood Samples

**Figure 1:**
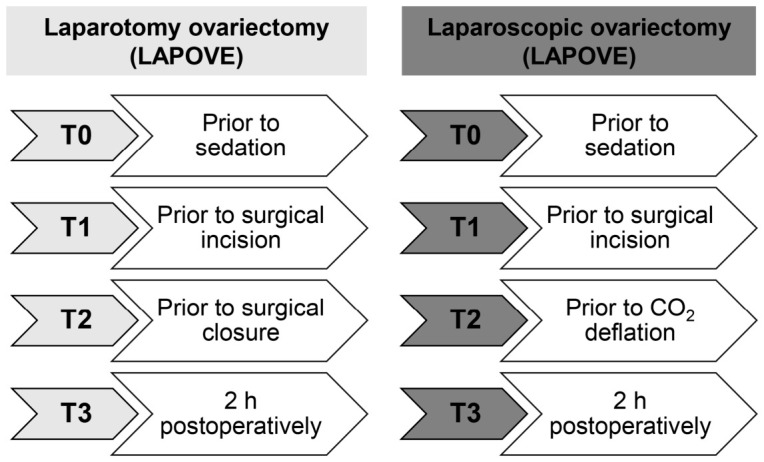
Timeline of blood sample collection.

Venous blood samples (2.0 mL per sample) were collected from the jugular vein into heparinized tubes. Samples were obtained at four predefined time points: T0 (baseline)—prior to sedation and drug administration; T1—immediately before surgical incision of the abdominal wall; T2—just before closure of the abdominal incision in the LPTOVE group and prior to CO_2_ desufflation in the LAPOVE group; and T3—2 h postoperatively (Figure 1). Plasma was separated by centrifugation at 1500 rpm for 15 min. For T0 and T3 time points, plasma was centrifuged within 30 min of sample collection, whereas for T1 and T2, centrifugation was performed immediately after sample collection. A volume of 100 µL of plasma per sample was used for the measurement of cortisol and total CO_2_ (TCO_2_) concentrations. The remaining plasma was stored at −80 °C until analysis of total oxidant status (TOS), total antioxidant status (TAS), and cytokines (TNF-α and IL-6) for no longer than 6 months [49]. Samples were thawed only once immediately prior to analysis, and no hemolysis was observed.

#### 2.4.1. Determination of Cortisol and Total CO_2_

Cortisol concentrations were measured using an automated immunoassay analyzer (AIA-360, Tosoh Corporation, Mumbai, India). The reference range for cortisol was 0.2–5.9 µg dL^−1^. TCO_2_ concentrations were determined at the T1 and T2 time points in dogs from the LPTOVE (*n* = 10) and LAPOVE (*n* = 10) groups using an automated clinical chemistry analyzer (DRI-CHEM NX600, FUJIFILM Corporation, Tokyo, Japan). The reference range for TCO_2_ was 16.00–26.00 mmol L^−1^.

#### 2.4.2. Determination of Oxidative Stress Parameters

TOS and TAS were determined using a Tecan Infinite^®^ 200 PRO plate reader (Tecan Trading AG, Männedorf, Switzerland) and colorimetric assay kits (Thermo Fisher Scientific, Waltham, MA, USA), following the manufacturer’s instructions [50,51]. Samples were analyzed in duplicate. The TOS assay had a sensitivity of 2.5 μmol H_2_O_2_ equivalents L^−1^, with intra- and inter-assay coefficients of variation of 2.3% and 3.5%, respectively [51]. The TAS assay had a sensitivity of 0.23 mmol Trolox equivalents L^−1^, with corresponding coefficients of variation of 4.6% and 7.0% [50].

TOS was measured using a colorimetric assay based on the oxidation of a chromogenic substrate. Briefly, 20 μL of plasma was mixed with 200 μL of chromogenic agent, and the initial absorbance at 590 nm was recorded (A_1_). After the addition of 50 μL of substrate solution, samples were incubated at 37 °C for 5 min, and absorbance was measured again (A_2_). The change in absorbance (ΔA = A_2_ − A_1_) was used for TOS calculation. The standard curve was generated using serial dilutions of a 200 μmol L^−1^ H_2_O_2_ stock solution to obtain final concentrations of 0–100 μmol L^−1^. The assay showed good linearity (R^2^ = 0.9764). TOS values were calculated as follows: TOS (μmol H_2_O_2_ Equiv. L^−1^) = (ΔA_sample − ΔA_blank − b) ÷ a × f, where a and b represent the slope and intercept of the calibration curve, respectively, and f is the dilution factor [16,51].

TAS was measured using a colorimetric assay based on the oxidation of ABTS (2,2′-azino-bis(3-ethylbenzothiazoline-6-sulfonic acid)). Briefly, 10 μL of plasma was mixed with 200 μL of buffer, and the initial absorbance was recorded at 660 nm (A_1_). After the addition of 20 μL of chromogenic agent, samples were mixed, incubated at 37 °C for 5 min, and the absorbance was measured again (A_2_). The change in absorbance (ΔA = A_2_ − A_1_) was used for TAS calculation. The standard curve was prepared using a 2 mmol L^−1^ Trolox stock solution serially diluted in 60% ethanol to obtain final concentrations of 0–2 mmol L^−1^. The assay showed excellent linearity (R^2^ = 0.9956). TAS values were calculated as follows: TAS (mmol Trolox Equiv. L^−1^) = (ΔA_blank − ΔA_sample − b) ÷ a × f, where a and b are the slope and intercept of the calibration curve, respectively, and f is the dilution factor [16,50].

The oxidative stress index (OSI) was calculated as follows: OSI (arbitrary unit) = TOS (μmol H_2_O_2_ Equiv. L^−1^)/TAS x 10 (mmol Trolox Equiv. L^−1^) [52].

#### 2.4.3. Determination of Cytokines (TNF-α and IL-6)

The concentrations of TNF-α and IL-6 were determined using commercially available canine ELISA kits (Thermo Fisher Scientific, Waltham, MA, USA), according to the manufacturer’s instructions [53,54]. The minimum detectable doses were 2 pg mL^−1^ for TNF-α and 100 pg mL^−1^ for IL-6. The intra- and inter-assay coefficients of variation for both assays were <10% and <12%, respectively [53,54].

Samples were diluted 1:2 in sample diluent. Briefly, 100 μL of diluted samples and standards were added in duplicate to 96-well microplates pre-coated with canine TNF-α and IL-6 antibodies and incubated for 2.5 h at room temperature. After incubation, wells were washed four times with wash buffer, followed by the addition of 100 μL of biotin-conjugated antibody and incubation for 1 h at room temperature. After washing, 100 μL of streptavidin–HRP solution was added to each well and incubated for 45 min at room temperature. Wells were washed again, and 100 μL of TMB substrate was added and incubated for 30 min at room temperature in the dark. The reaction was stopped by adding 50 μL of stop solution, and the optical density (OD) was measured at 450 nm using a Tecan Infinite^®^ 200 PRO plate reader (Tecan Trading AG, Männedorf, Switzerland) [53,54]. TNF-α and IL-6 concentrations were calculated from standard curves generated using the standards provided in the kits, according to the manufacturer’s instructions, with coefficients of determination (R^2^) of 0.9913 and 0.9299, respectively.

### 2.5. Statistical Analysis

Data normality was assessed using the Shapiro–Wilk test. As most variables did not follow a normal distribution, the results are presented as the median and interquartile range [IQR: 25th–75th percentiles]. Statistical analyses were conducted using the Mann–Whitney U and Wilcoxon Signed Rank tests. A *p* value of < 0.05 was considered statistically significant. All statistical calculations were performed using IBM SPSS Statistics 31.0.0.0 (IBM Corp., Armonk, NY, USA).

## 3. Results

Both the LAPOVE and LPTOVE groups were matched for age (24 [IQR: 12–68] and 21 [IQR: 12–24] months, respectively) and body weight (12.9 [IQR: 6.9–17.3] and 10.4 [IQR: 8.0–19.5] kg, respectively). The laparoscopic OVE procedure was 5 min shorter than the laparotomy OVE procedure (55 [IQR: 50–55] and 60 [IQR: 55–65] min, respectively) (*p* = 0.003). Similarly, the total anesthesia time (T0–T3) was 5 min shorter in the LAPOVE group than in the LPTOVE group (85 [IQR: 80–85] and 90 [IQR: 85–95] min, respectively) (*p* = 0.001). Baseline physiological and vital parameters measured before the procedure did not differ significantly between the groups. All dogs remained stable throughout the procedure and completed the study.

### 3.1. Cortisol Evaluation

**Figure 2:**
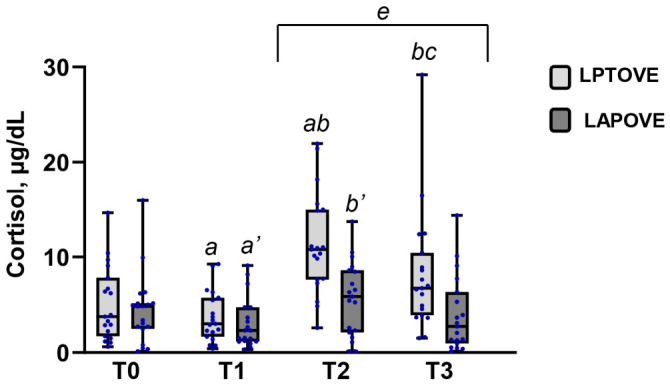
Plasma cortisol concentration changes in healthy dogs undergoing open and laparoscopic ovariectomy at different study time points. Data are presented as median and interquartile range [IQR]. In the boxplot, the central line represents the median, the box indicates the interquartile range [25th–75th percentile], whiskers represent the minimum and maximum values, and dots represent individual values. (T0)—blood sample taken prior to sedation; (T1)—prior to surgical incision; (T2)—prior to closure of the abdominal incision in the LPTOVE group, and prior to CO_2_ desufflation in the LAPOVE group; (T3)—blood sample taken 2 h postoperatively. *a* indicates significant differences from T0 within the LPTOVE group (*p* < 0.05). *b* indicates significant differences from T1 within the LPTOVE group (*p* < 0.05). *c* indicates significant differences from T2 within the LPTOVE group (*p* < 0.05). *a’* indicates significant differences from T0 within the LAPOVE group (*p* < 0.05). *b’* indicates significant differences from T1 within the LAPOVE group (*p* < 0.05). *e* indicates significant differences between the LPTOVE and LAPOVE groups at T2 and T3 time points (*p* < 0.05).

Plasma cortisol concentration was assessed at four time points (T0–T3) in both animal groups (Figure 2). In the LPTOVE group, the cortisol concentration decreased significantly from T0 (3.73 [IQR: 1.69–7.81] µg dL^−1^) to T1 (2.98 [IQR: 1.64–5.70] µg dL^−1^) (*p* = 0.035). In contrast, cortisol levels at T2 (10.76 [IQR: 7.62–14.95] µg dL^−1^) increased significantly by 189% and 261% compared with T0 and T1, respectively (both *p* = 0.001). At 2 h after surgery (T3), cortisol concentration decreased significantly to 6.71 [IQR: 3.89–10.40] µg dL^−1^, representing a 38% decrease compared with T2, while remaining 125% higher than T1 (*p* = 0.025, *p* = 0.004, respectively). In addition, at T2 and T3, cortisol concentrations exceeded the upper limit of the reference range by 82% and 14%, respectively. In the LAPOVE group, the cortisol concentration decreased significantly from T0 (4.81 [IQR: 2.47–5.13] µg dL^−1^) to T1 (2.29 [IQR: 1.14–4.72] µg dL^−1^) (*p* = 0.011). However, at T2 (5.83 [IQR: 2.08–8.59] µg dL^−1^), cortisol concentration increased significantly by 155% compared with T1 (*p* = 0.019). All values remained within the reference range. Between-group analysis showed higher cortisol levels in the LPTOVE group at T2 (10.76 [IQR: 7.62–14.95] and 5.83 [IQR: 2.08–8.59] µg dL^−1^, respectively) and T3 (6.71 [IQR: 3.89–10.40] and 2.71 [IQR: 0.95–6.31] µg dL^−1^, respectively), corresponding to 85% and 148% increases compared with the LAPOVE group (*p* = 0.001, *p* = 0.006, respectively).

### 3.2. Oxidative Stress Evaluation

**Figure 3:**
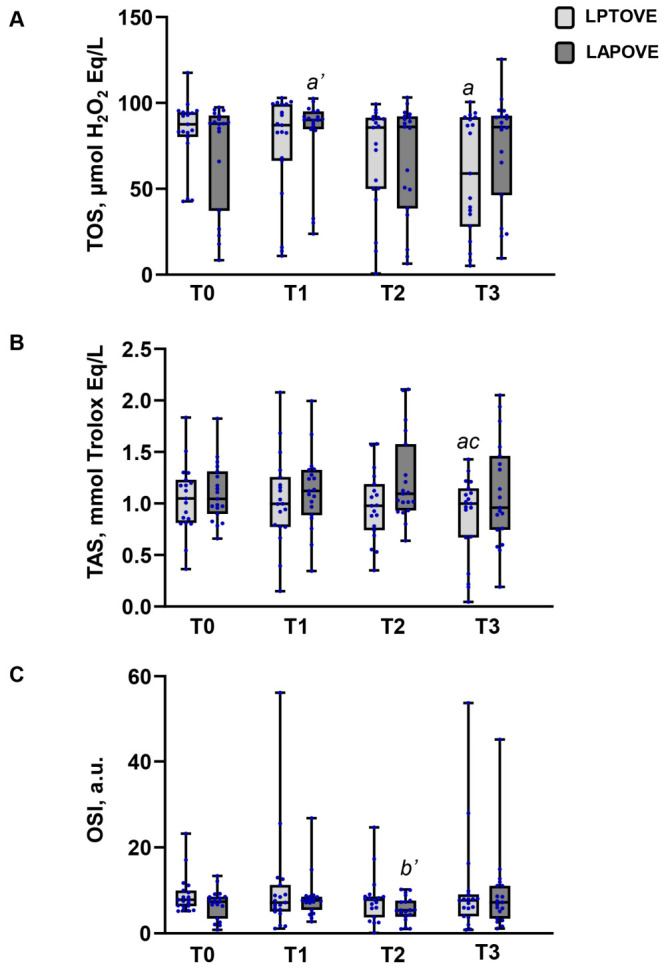
OS changes in healthy dogs undergoing open and laparoscopic ovariectomy at different study time points: (**A**) total oxidant status (TOS), (**B**) total antioxidant status (TAS), (**C**) oxidative stress index (OSI). Data are presented as median and interquartile range [IQR]. In the boxplot, the central line represents the median, the box indicates the interquartile range [25th–75th percentile], whiskers represent the minimum and maximum values, and dots represent individual values. (T0)—blood sample taken prior to sedation; (T1)—prior to surgical incision; (T2)—prior to closure of the abdominal incision in the LPTOVE group, and prior to CO_2_ desufflation in the LAPOVE group; (T3)—blood sample taken 2 h postoperatively. *a* indicates significant differences from T0 within the LPTOVE group (*p* < 0.05). *c* indicates significant differences from T2 within the LPTOVE group (*p* < 0.05). *a’* indicates significant differences from T0 within the LAPOVE group (*p* < 0.05). *b’* indicates significant differences from T1 within the LAPOVE group (*p* < 0.05).

Changes in oxidative stress parameters (TOS, TAS, and OSI) following the LPTOVE and LAPOVE procedures at different time points (T0–T3) are presented in Figure 3. In the LPTOVE group, TOS decreased significantly at T3 (58.87 [IQR: 27.81–91.51] μmol H_2_O_2_ Equiv. L^−1^), representing a 33% reduction compared to T0 (87.39 [IQR: 80.12–94.44] μmol H_2_O_2_ Equiv. L^−1^) (*p* = 0.005). Similarly, TAS decreased significantly at T3 (1.00 [IQR: 0.67–1.14] mmol Trolox Equiv. L^−1^), representing a 5% reduction compared to T0 (1.05 [IQR: 0.81–1.22] mmol Trolox Equiv. L^−1^) (*p* = 0.016). In contrast, TAS increased significantly from T2 (0.98 [IQR: 0.74–1.18] mmol Trolox Equiv. L^−1^) to T3 (1.00 [IQR: 0.67–1.14] mmol Trolox Equiv. L^−1^) (*p* = 0.040). No significant differences in OSI were observed across the T0–T3 time points. In the LAPOVE group, TOS increased significantly from T0 (87.84 [IQR: 37.00–92.59] μmol H_2_O_2_ Equiv. L^−1^) to T1 (90.00 [IQR: 84.57–94.88] μmol H_2_O_2_ Equiv. L^−1^) (*p* = 0.021). However, OSI decreased significantly at T2 (5.35 [IQR: 3.81–7.60] arbitrary units), representing a 30% reduction compared to T1 (7.61 [IQR: 5.43–8.47] arbitrary units) (*p* = 0.049). TAS changes were mild and non-significant across all four time points. No significant differences in TOS, TAS, and OSI levels were observed between the LPTOVE and LAPOVE groups across the T0–T3 time points.

### 3.3. Cytokine Evaluation

TNF-α and IL-6 levels were assessed at the T0–T3 time points in the LPTOVE and LAPOVE groups. TNF-α and IL-6 levels were below the detection limit at all time points in both groups (Table 1).

### 3.4. Evaluation of Total CO_2_

**Figure 4:**
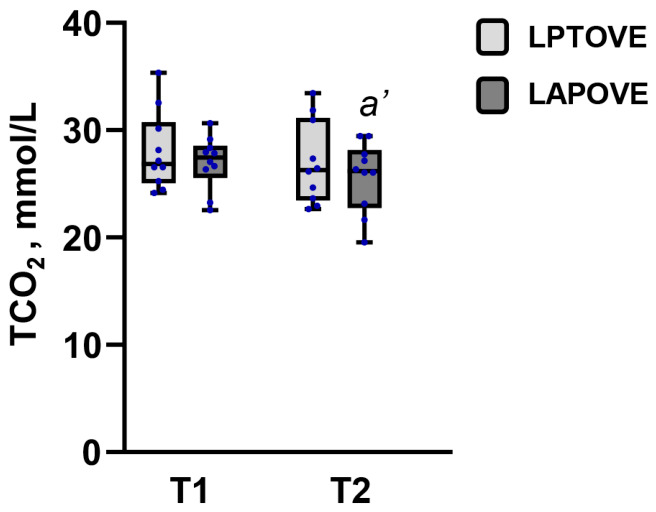
Plasma total CO_2_ (TCO2) changes in healthy dogs undergoing open and laparoscopic ovariectomy at different study time points. Data are presented as median and interquartile range [IQR]. In the boxplot, the central line represents the median, the box indicates the interquartile range [25th–75th percentile], whiskers represent the minimum and maximum values, and dots represent individual values. (T1)—prior to surgical incision; (T2)—prior to closure of the abdominal incision in the LPTOVE group, and prior to CO_2_ desufflation in the LAPOVE group. *a’* indicates significant differences from T1 within the LAPOVE group (*p* < 0.05).

Plasma total CO_2_ (TCO_2_) concentration was assessed at T1 and T2 time points (Figure 4). In the LPTOVE group, no significant changes were observed. In the LAPOVE group, TCO_2_ decreased significantly at T2 (26.15 [IQR: 23.10–28.90] mmol L^−1^), representing a 5% reduction compared to T1 (27.40 [IQR: 26.30–29.10] mmol L^−1^) (*p* = 0.037). TCO_2_ values slightly exceeded the upper reference limit at both time points in both groups. In the LPTOVE group, values were 3% and 1% above the upper reference limit at T1 and T2, respectively, while in the LAPOVE group the corresponding increases were 5% and 0.6%. No significant differences in TCO_2_ concentration were observed between the LPTOVE and LAPOVE groups at either time point.

### 3.5. Respiratory Parameters Evaluation

**Figure 5:**
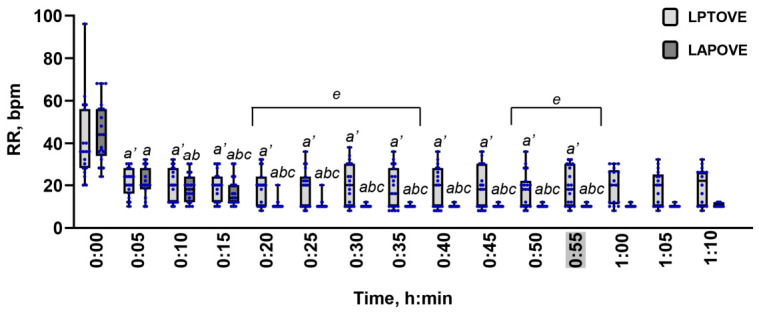
Changes in respiratory rate (RR) in healthy dogs undergoing open and laparoscopic ovariectomy at different study time points. Data are presented as median and interquartile range [IQR]. In the boxplot, the central line represents the median, the box indicates the interquartile range [25th–75th percentile], whiskers represent the minimum and maximum values, and dots represent individual values. The analysis endpoint (t = 55 min) is shown as a gray-shaded area. *a’* indicates significant differences from t = 0 min within the LPTOVE group (*p* < 0.05). *a* indicates significant differences from t = 0 min within the LAPOVE group (*p* < 0.05). *b* indicates significant differences from t = 5 min within the LAPOVE group (*p* < 0.05). *c* indicates significant differences from t = 10 min within the LAPOVE group (*p* < 0.05). *e* indicates significant differences between the LPTOVE and LAPOVE groups (*p* < 0.05).

**Figure 6:**
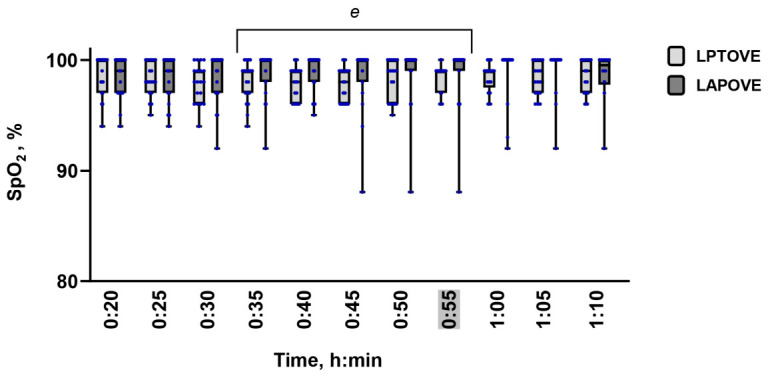
Changes in oxygen saturation (SpO_2_) in healthy dogs undergoing open and laparoscopic ovariectomy at different study time points. Data are presented as median and interquartile range [IQR]. In the boxplot, the central line represents the median, the box indicates the interquartile range [25th–75th percentile], whiskers represent the minimum and maximum values, and dots represent individual values. The analysis endpoint (t = 55 min) is shown as a gray-shaded area. *e* indicates significant differences between the LPTOVE and LAPOVE groups (*p* < 0.05).

**Figure 7:**
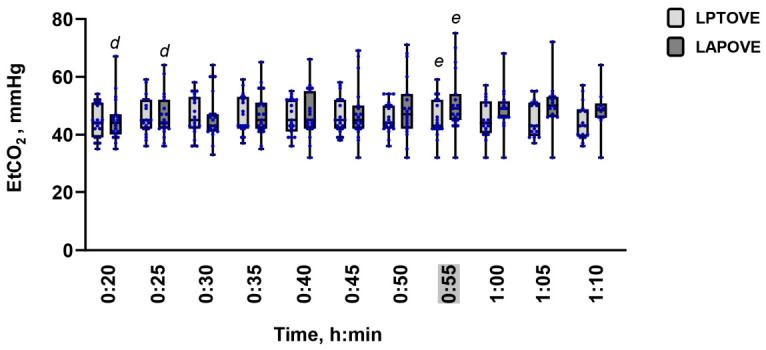
Changes in end-tidal CO_2_ (EtCO_2_) in healthy dogs undergoing open and laparoscopic ovariectomy at different study time points. Data are presented as median and interquartile range [IQR]. In the boxplot, the central line represents the median, the box indicates the interquartile range [25th–75th percentile], whiskers represent the minimum and maximum values, and dots represent individual values. The analysis endpoint (t = 55 min) is shown as a gray-shaded area. *d* indicates significant differences from t = 55 min within the LAPOVE group (*p* < 0.05). *e* indicates significant differences between the LPTOVE and LAPOVE groups (*p* < 0.05).

RR, SpO_2_, and EtCO_2_ values are presented in Figure 5, Figure 6 and Figure 7. RR was continuously monitored starting before sedation (t = 0 min), whereas SpO_2_ and EtCO_2_ were recorded from 20 min after sedation (t = 20 min) until the end of anesthesia. Due to heterogeneous procedure durations and insufficient sample size at later time points, the analysis endpoint was defined at t = 55 min.

In the LPTOVE group, RR decreased significantly from t = 5 min (24 [IQR: 16–28] bpm) and remained significantly lower at all subsequent time points up to t = 55 min (18 [IQR: 10–30] bpm) compared with t = 0 min (36 [IQR: 28–56] bpm) (*p* < 0.05) (Figure 5). In the LAPOVE group, RR decreased significantly at t = 5 min (20 [IQR: 18–28] bpm) compared with t = 0 min (44 [IQR: 34–56] bpm) (*p* = 0.001). Further significant reductions were observed at t = 10 min (18 [IQR: 12–24] bpm) and t = 15 min (14 [IQR: 12–20] bpm) compared with t = 5 min and t = 10 min, respectively (*p* = 0.005, *p* = 0.001, respectively). RR remained significantly lower at all subsequent time points up to t = 55 min (10 [IQR: 10-10] bpm) compared with the early phase (t = 0–15 min) (*p* < 0.05). The comparative analysis showed higher RR in the LPTOVE group from t = 20 min (20 [IQR: 10–24] bpm) to t = 35 min (16 [IQR: 10–28] bpm) compared with the LAPOVE group at the same time points (10 [IQR: 10–10] bpm at both time points) (*p* < 0.05). Similarly, RR in the LPTOVE group was higher at the last two time points (t = 50 min; 18 [IQR: 10–22] bpm and t = 55 min; 18 [IQR: 10–30] bpm) compared with the LAPOVE group (10 [IQR: 10–10] bpm at both time points) (both *p* = 0.001).

No significant differences in SpO_2_ were observed within the LPTOVE and LAPOVE groups between t = 20 min and t = 55 min (Figure 6). However, SpO_2_ was significantly higher in the LAPOVE group from t = 35 min (100 [IQR: 98–100] %) to t = 55 min (100 [IQR: 99–100] %) compared with the LPTOVE group at the same time points (99 [IQR: 97–99] % at both time points) (*p* < 0.05).

No significant differences in EtCO_2_ were observed within the LPTOVE group (Figure 7). In the LAPOVE group, EtCO_2_ at t = 20 min (44 [IQR: 40–47] mmHg) and t = 25 min (44 [IQR: 42–52] mmHg) was significantly lower compared with t = 55 min (49 [IQR: 45–54] mmHg) (*p* = 0.013, *p* = 0.006, respectively). At t = 55 min, EtCO_2_ was higher in the LAPOVE group (49 [IQR: 45–54] mmHg), representing a 14% increase compared with the LPTOVE group (43 [IQR: 42–52] mmHg) (*p* = 0.038).

## 4. Discussion

Previous studies have extensively compared laparoscopic and open OVE procedures. However, to the best of our knowledge, this is the first study to provide an integrated assessment of cortisol, OS, inflammatory, and systemic CO_2_ responses in healthy female dogs undergoing open and laparoscopic OVE during the intraoperative and early postoperative periods, while systemic CO_2_ responses were evaluated only intraoperatively.

Data regarding intraoperative cortisol responses in dogs undergoing open and laparoscopic OVE remain limited. In our previous study, plasma cortisol concentrations measured at T2 (immediately before abdominal closure in the LPTOVE group and before CO_2_ desufflation in the LAPOVE group) tended to be higher in dogs undergoing open OVE than in those undergoing laparoscopic OVE. However, this difference did not reach statistical significance, likely owing to the limited sample size [16]. In contrast, the present study demonstrated that cortisol levels at T2 were 85% higher in the LPTOVE group compared with the LAPOVE group. Previous studies have largely focused on postoperative changes in plasma cortisol concentrations and have demonstrated higher cortisol concentrations at 0.5, 1, 2, 3, and 6 h after open OVE or OHE than after the corresponding laparoscopic procedures [32,33,36,37]. Our previous study, which included a smaller sample size, also reported significantly higher cortisol levels 2 h after open OVE than after laparoscopic OVE [16]. These observations were further supported by the present study, in which cortisol concentrations at 2 h postoperatively (T3) were significantly higher in the LPTOVE group than in the LAPOVE group. Specifically, plasma cortisol levels at T3 were 148% higher in the LPTOVE group than in the LAPOVE group and exceeded the upper limit of the reference range. In both groups, cortisol levels decreased at T1 compared with T0. These findings differ from previous studies reporting that general anesthesia alone does not significantly reduce plasma cortisol concentrations in dogs compared with awake controls [55,56]. Although data regarding the effects of general anesthesia on cortisol dynamics remain limited, one possible explanation for the observed reduction in cortisol levels before surgical incision is the suppressive effect of α_2_-adrenergic agonists and opioids on the hypothalamic–pituitary–adrenal axis. These agents may attenuate stress-induced cortisol secretion through reduced adrenocorticotropic hormone (ACTH) release [57,58,59,60]. Nevertheless, further studies are needed to elucidate the mechanisms underlying this observation. Following this initial decrease, cortisol concentrations increased significantly from T1 to T2 in both groups. Moreover, in the LPTOVE group, cortisol levels exceeded the upper limit of the reference range by 82%, while remaining within the reference range in the LAPOVE group. The observed differences in stress-related responses suggest that open OVE imposes a greater physiological burden than laparoscopic OVE both towards the end of surgery and during the early postoperative period. This difference is likely attributable to the greater degree of tissue manipulation and surgical trauma associated with the open technique, while the minimally invasive laparoscopic approach may mitigate the surgical stress response.

Previous studies have consistently reported lower postoperative oxidative stress following laparoscopic OVE/OHE compared with the corresponding open procedures [11,26,27,29,31]. Specifically, Lee et al. reported higher TOS values immediately after surgery in the open OVE group [11], whereas other studies detected increased oxidative stress at 2 h, 96 h, and 168 h postoperatively [27,29,31]. The present findings differ from those reported in previous studies. No significant between-group differences were detected in TOS, TAS, or OSI, indicating broadly comparable oxidative stress responses following both surgical procedures. Furthermore, TOS decreased by 33% at T3 relative to baseline in the LPTOVE group, whereas it remained largely unchanged in the LAPOVE group, apart from a mild transient increase at T1. Similarly, TAS exhibited minor fluctuations in the LPTOVE group but remained stable in the LAPOVE group. The limited changes observed in TAS may also reflect the buffering capacity of circulating plasma antioxidants, including albumin and other endogenous antioxidant molecules, which contribute substantially to the antioxidant capacity measured by ABTS-based assays. Consequently, mild to moderate surgical trauma may not be sufficient to produce detectable changes in systemic TAS despite transient alterations in oxidative status [61,62]. In addition, the absence of marked postoperative oxidative stress changes may be partly explained by the perioperative management applied in the present study, as appropriate anesthetic and analgesic protocols have been shown to attenuate surgery-induced oxidative injury in dogs [23]. Perioperative redox homeostasis reflects the dynamic interaction between surgery-induced oxidative stress and the effects of anesthetic and analgesic agents on endogenous antioxidant defense mechanisms [20]. As all dogs received the same standardized multimodal anesthetic and analgesic protocol, the combined effects of this protocol, together with adequate perioperative pain control, may have contributed to maintaining redox homeostasis by limiting the systemic oxidative response to surgical trauma. However, because the present study did not include an anesthesia-only control group and was not designed to evaluate the independent effects of anesthesia on oxidative stress, the contribution of the anesthetic protocol cannot be distinguished from that of surgical trauma. Further studies are warranted to clarify the individual effects of anesthetic agents on perioperative redox homeostasis.

The interpretation of these findings should also be considered in relation to the methodological scope of the selected oxidative stress biomarkers. TOS, TAS, and OSI were selected in accordance with the primary objective of the present study, which was to compare the overall systemic oxidative response following open and laparoscopic OVE rather than to characterize specific molecular pathways of oxidative damage. These biomarkers provide an integrated assessment of systemic oxidant–antioxidant balance and were therefore considered appropriate for evaluating the systemic effects of perioperative tissue trauma in this clinical setting. In addition, the available plasma volume obtained from serial blood sampling required prioritization of the planned analyses (cortisol, TOS, TAS, TCO_2_, TNF-α, and IL-6), precluding the inclusion of additional biomarkers of oxidative damage, such as malondialdehyde (MDA), a marker of lipid peroxidation, and 8-hydroxy-2′-deoxyguanosine (8-OHdG), a marker of oxidative DNA damage. Although these biomarkers would have provided valuable mechanistic information regarding specific oxidative damage pathways, the selected markers of systemic oxidant–antioxidant balance were considered sufficient for addressing the primary objective of the study by comparing the overall systemic oxidative response induced by the two surgical approaches. Nevertheless, the present findings should be interpreted as reflecting overall redox homeostasis rather than specific molecular mechanisms of oxidative injury.

Additionally, oxidative stress represents a highly dynamic process, and the selected sampling time points may not have captured the peak postoperative oxidative stress response [63,64]. The observed decrease in TOS may reflect dynamic and transient alterations in redox homeostasis, potentially driven by endogenous antioxidant responses and inter-individual variability [49,65,66]. Therefore, the biological relevance of this finding should be interpreted with caution. Importantly, these changes in TOS and TAS were not accompanied by corresponding changes in OSI, which remained stable throughout the study period in the LPTOVE group. Conversely, in the LAPOVE group, TOS and TAS remained largely unchanged, while OSI decreased by 30% at T2 compared with T1. These observations may be related to subtle changes in the relative balance between oxidant and antioxidant status that were not apparent when each parameter was evaluated separately. As OSI represents a ratio of TOS to TAS, it may be more sensitive to minor fluctuations in these variables. Alternatively, these observations may reflect biological and analytical variability rather than a consistent effect of the surgical procedure.

Interestingly, in both the present study and our previous investigation, serum cortisol concentrations were significantly higher in dogs undergoing open OVE than in those undergoing laparoscopic OVE at the end of surgery and 2 h postoperatively, whereas no corresponding between-group differences were detected in TOS, TAS, or OSI [16]. Although the present study was not designed to evaluate the relative performance of different surgical stress biomarkers, these findings may suggest that cortisol was more responsive than the evaluated oxidative stress markers to differences in tissue trauma during the early postoperative period. However, the temporal patterns of endocrine and oxidative stress responses may differ substantially, and the selected sampling time points may not have captured peak oxidative stress changes. Therefore, this observation should be interpreted with caution and warrants further investigation.

Only a limited number of studies have evaluated inflammatory responses to tissue trauma in dogs undergoing open and laparoscopic OVE, and most have reported higher postoperative inflammatory marker concentrations in dogs undergoing open OVE [28,31,37]. Only two studies have evaluated the pro-inflammatory cytokines IL-6 and TNF-α in dogs undergoing open and laparoscopic OVE. Okur et al. reported significantly higher postoperative IL-6 and TNF-α concentrations in the open OVE group at 1, 3, and 6 h after surgery [37]. Calabria et al. reported higher IL-6 concentrations in the open OVE group at baseline, 24 h, and 7 days postoperatively, while dogs undergoing laparoscopic OVE exhibited only a transient increase in IL-6 concentrations at 2 h after surgery [31]. In contrast to previous reports, IL-6 and TNF-α concentrations remained below the detection limit throughout the study period in both groups. Elective ovariectomy in healthy dogs has been associated with a relatively mild to moderate systemic inflammatory response compared with more invasive surgical procedures [31,67]. Therefore, postoperative circulating cytokine concentrations may remain relatively low and may be more difficult to detect, particularly following minimally invasive procedures or when mild tissue injury is involved. In the present study, commercially available canine-specific ELISA kits were used, with analytical sensitivities of 100 pg/mL for IL-6 and 2 pg/mL for TNF-α [53,54]. Consequently, subtle postoperative increases in circulating cytokine concentrations, particularly IL-6, may have remained below the analytical detection limits of the assays. Furthermore, the absence of detectable IL-6 and TNF-α concentrations may be related to the selected sampling schedule, which may not have coincided with peak postoperative cytokine release. Previous studies have shown that circulating IL-6 concentrations in dogs may increase several hours after tissue injury and reach peak values between 4 and 12 h postoperatively, whereas TNF-α appears to peak earlier, approximately 60 min after injury [68,69]. Additionally, the multimodal anesthetic and analgesic protocol used in the present study may have attenuated perioperative inflammatory responses. Although evidence in dogs is limited, studies in human patients have suggested that dexmedetomidine may attenuate perioperative inflammatory responses by reducing circulating IL-6 and TNF-α concentrations [70,71]. Therefore, the contribution of dexmedetomidine to the absence of detectable cytokine responses in the present study cannot be excluded. Overall, the absence of detectable IL-6 and TNF-α concentrations should be interpreted with caution, as it may reflect the combined influence of biological response magnitude, assay sensitivity, sampling timing, and perioperative management.

Systemic carbon dioxide status was assessed by measuring plasma total CO_2_ (TCO_2_) at T1 and T2, and monitoring end-tidal CO_2_ (EtCO_2_) during anesthesia. Fukushima et al. reported that CO_2_ insufflation during laparoscopic surgery may result in systemic CO_2_ absorption, leading to hypercapnia, hypoventilation and respiratory acidosis [15]. In the present study, no significant differences in TCO_2_ values were observed between groups. However, a mild reduction at T2 compared with T1 was observed in the LPTOVE group. In both groups, TCO2 values were slightly above the upper reference limit at both time points, with deviations not exceeding 5%. These findings may suggest that both surgical approaches were associated with only minimal and clinically irrelevant alterations in systemic carbon dioxide balance. The slight decrease observed in the LPTOVE group at T2 may reflect transient changes in CO_2_ kinetics associated with PNP and its physiological compensation during anesthesia [12,15]. However, the absence of marked alterations in either group may indicate effective ventilatory management and adequate maintenance of acid–base homeostasis.

Previous studies have demonstrated that EtCO_2_ remains relatively stable throughout laparoscopic OVE in dogs, showing only minor variations related to surgical stage [29,72,73]. In the present study, EtCO_2_ values were significantly higher at the endpoint of analysis (t = 55 min) in the LAPOVE group compared with the LPTOVE group. A similar pattern was observed in our previous study, where EtCO_2_ was higher at the last three anesthesia time points in the laparoscopic OVE group compared with the open OVE group [16]. These findings likely reflect transient CO_2_ absorption associated with pneumoperitoneum during laparoscopy. However, controlled mechanical ventilation using a volume-controlled mode during laparoscopic OVE may have supported respiratory compensation for increased CO_2_ absorption associated with pneumoperitoneum by allowing maintenance of adequate alveolar ventilation [12]. During pneumoperitoneum, absorbed CO_2_ increases the amount of CO_2_ delivered to the circulation. Therefore, maintaining sufficient minute ventilation is essential to prevent excessive increases in circulating CO_2_ concentrations [12,15]. In the present study, mechanical ventilation may have contributed to this compensatory response by allowing controlled adjustment of ventilatory parameters throughout the procedure. In contrast, dogs undergoing open OVE were allowed to breathe spontaneously, and their ability to increase ventilation in response to increased CO_2_ production depended on their physiological respiratory response. The absence of between-group differences in TCO_2_ may indicate that these mechanisms contributed to maintaining relatively stable CO_2_ status during the procedure.

To further evaluate respiratory function during anesthesia, respiratory rate (RR) and peripheral oxygen saturation (SpO_2_) were also assessed. Several previous studies have reported no significant between-group differences in RR or SpO_2_ in dogs undergoing open and laparoscopic OVE [26,29,72,73]. In the present study, RR decreased significantly in both groups shortly after sedation and remained lower throughout the procedure. This finding may be partly explained by the sedation protocol used, which consisted of methadone, a full μ-opioid receptor agonist, and dexmedetomidine, an α_2_-adrenergic receptor agonist. Previous studies have demonstrated that this drug combination is associated with moderate reductions in respiratory rate in dogs undergoing anesthesia and surgery [74,75,76]. Nevertheless, significant between-group differences in RR were observed at several time points during anesthesia, with lower RR values recorded in the LAPOVE group. One possible explanation for this finding is the different ventilatory approach between groups, as respiratory rate in the LAPOVE group was determined by ventilator settings under volume-controlled ventilation, whereas in the LPTOVE group it reflected spontaneous breathing. Accordingly, RR may have been influenced by ventilatory management rather than solely by the physiological effects of surgery. Similarly, SpO_2_ values were significantly higher in the LAPOVE group between t = 35 and 55 min. This finding may also reflect differences in ventilatory management between groups. However, median SpO_2_ values exceeded 98% in both groups throughout the study period, suggesting that oxygenation remained clinically adequate regardless of the surgical approach.

This study has several limitations. First, no a priori sample size calculation or statistical power analysis was performed. Consequently, the study may have been underpowered to detect differences in several outcome variables, including TOS, TAS, OSI, TNF-α, and IL-6. Therefore, the false-negative findings cannot be excluded. Second, allocation to the study groups was based on owner preference rather than random assignment. Although baseline characteristics were comparable between groups, this non-random allocation may have introduced selection bias and residual confounding, limiting the ability to attribute the observed differences solely to the surgical technique. Furthermore, potential environmental and multifactorial influences on oxidant and antioxidant status were not assessed, and reproductive history was not recorded. Therefore, possible effects on hormonal balance and oxidative stress responses cannot be excluded. The distribution of breeds differed between groups, with a higher proportion of purebred dogs in the LAPOVE group. Although body weight was comparable between groups, breed-related differences in metabolism and antioxidant capacity may have contributed to inter-individual variability in oxidative status parameters. Consequently, a potential influence of breed heterogeneity on redox-related outcomes cannot be completely excluded. In addition, the LAPOVE group had a 5 min shorter surgical and anesthetic duration, which represents a minor methodological difference between groups and is unlikely to have influenced the study outcomes. Moreover, oxidative status was assessed using systemic markers of oxidant–antioxidant balance, including TOS, TAS, and OSI. Although these parameters provide an overall assessment of systemic redox status, they do not directly assess specific molecular oxidative damage pathways or cellular antioxidant defense mechanisms. The inclusion of additional biomarkers, such as MDA, 8-OHdG, and enzymatic antioxidants (superoxide dismutase, catalase, and glutathione peroxidase), could have provided complementary mechanistic insight into oxidative injury associated with surgical trauma. Furthermore, the inflammatory response was assessed only by measuring TNF-α and IL-6 during the first 2 h after surgery. Therefore, a delayed postoperative inflammatory response cannot be excluded. Finally, systemic carbon dioxide status was evaluated only using TCO_2_ and EtCO_2_, while arterial blood gas parameters such as pH and PaCO_2_ were not measured, limiting a more precise assessment of acid–base changes associated with pneumoperitoneum.

Future studies should investigate the potential effects of anesthetic and analgesic protocols on oxidative stress, as these factors may contribute to perioperative redox modulation. In addition, extended postoperative sampling is warranted to better characterize the temporal dynamics of oxidative stress following surgical trauma. Furthermore, future investigations should combine systemic oxidative stress indices with specific biomarkers of oxidative damage, such as MDA and 8-OHdG, together with enzymatic antioxidants including superoxide dismutase, catalase, and glutathione peroxidase, to provide a more comprehensive understanding of the molecular mechanisms underlying oxidative injury and cellular antioxidant responses associated with surgical trauma and CO_2_ pneumoperitoneum. Systemic carbon dioxide status should also be evaluated more comprehensively using arterial blood gas analysis, including pH and PaCO_2_, to allow a more accurate assessment of acid–base changes associated with pneumoperitoneum. Finally, a more comprehensive assessment of perioperative stress associated with different surgical techniques may be achieved by combining validated pain assessment scales with cortisol measurements.

## 5. Conclusions

Laparoscopic ovariectomy was associated with lower perioperative cortisol concentrations compared with open surgery, whereas no significant differences were observed in oxidative stress markers (TOS, TAS, and OSI) or total CO_2_ dynamics. These findings suggest that laparoscopic OVE may be associated with a reduced early endocrine stress response under the conditions of the present study, while systemic redox status and carbon dioxide-related parameters remained largely comparable between surgical approaches.

## Figures and Tables

**Table 1:** Cytokines (TNF-α and Il-6) at different time points in dogs undergoing open (LPTOVE) and laparoscopic (LAPOVE).

	TNF-α, pg mL^−1^				IL-6, pg mL^−1^			
	T0	T1	T2	T3	T0	T1	T2	T3
LPTOVE	<2				<100			
LAPOVE	<2				<100			

(T0)—blood sample taken prior to sedation; (T1)—prior to surgical incision; (T2)—prior to closure of the abdominal incision in the LPTOVE group, and prior to CO_2_ desufflation in the LAPOVE group; (T3)—blood sample taken 2 h postoperatively.

## Data Availability

The data presented in this study are available on request from the corresponding author upon reasonable request.

## Abbreviations

The following abbreviations are used in this manuscript:

OVE	Ovariectomy
OHE	Ovariohysterectomy
PNP	Pneumoperitoneum
CO_2_	Carbon dioxide
IAP	Intra-abdominal pressure
LPTOVE	Laparotomy ovariectomy
LAPOVE	Laparoscopic ovariectomy
OS	Oxidative stress
TOS	Total oxidant status
TAS	Total antioxidant status
OSI	Oxidative stress index
IL-6	Interleukin 6
TNF-α	Tumor necrosis factor alpha
OD	Optical density
TCO_2_	Total CO_2_
RR	Respiratory rate
SpO_2_	Oxygen saturation
EtCO_2_	End-tidal CO_2_
ROS	Reactive oxygen species
MDA	Malondialdehyde
8-OHdG	8-hydroxy-2′-deoxyguanosine

## References

[1] Schwarzkopf I., Van Goethem B., Vandekerckhove P.M., de Rooster H. (2015). Vessel sealing versus suture ligation for canine ovarian pedicle haemostasis: A randomised clinical trial. Vet. Rec..

[2] Watts J. (2018). The use of bipolar electrosurgical forceps for haemostasis in open surgical ovariectomy of bitches and queens and castration of dogs. J. Small Anim. Pract..

[3] Urfer S.R., Kaeberlein M. (2019). Desexing dogs: A review of the current literature. Animals.

[4] Dupré G., Fiorbianco V., Skalicky M., Gültiken N., Ay S.S., Findik M. (2009). Laparoscopic ovariectomy in dogs: Comparison between single portal and two-portal access. Vet. Surg..

[5] Nylund A.M., Drury A., Weir H., Monnet E. (2017). Rates of intraoperative complications and conversion to laparotomy during laparoscopic ovariectomy performed by veterinary students: 161 cases (2010–2014). J. Am. Vet. Med. Assoc..

[6] Bakhtiari J., Khalaj A.R., Aminlou E., Niasari-Naslaji A. (2012). Comparative evaluation of conventional and transvaginal laparoscopic ovariohysterectomy in dogs. Vet. Surg..

[7] Corriveau K.M., Giuffrida M.A., Mayhew P.D., Runge J.J. (2017). Outcome of laparoscopic ovariectomy and laparoscopic-assisted ovariohysterectomy in dogs: 278 cases (2003–2013). J. Am. Vet. Med. Assoc..

[8] Cicirelli V., Lacalandra G.M., Aiudi G.G. (2022). The effect of splash block on the need for analgesia in dogs subjected to video-assisted ovariectomy. Vet. Med. Sci..

[9] Cassata G., Palumbo V.D., Cicero L., Damiano G., Maenza A., Migliazzo A., Di Paola G., Vicari D., Fazzotta S., Lo Monte A.I. (2016). Laparotomic vs laparoscopic ovariectomy: Comparing the two methods. The ovariectomy in the bitch in laparoscopic era. Acta Biomed..

[10] Di Bella C., Lacitignola L., Grasso S., Centonze P., Greco A., Ostuni R., Crovace A., Staffieri F. (2018). An alveolar recruitment maneuver followed by positive end-expiratory pressure improves lung function in healthy dogs undergoing laparoscopy. Vet. Anaesth. Analg..

[11] Kim M.C., Lee J.Y. (2014). Comparison of oxidative stress status in dogs undergoing laparoscopic and open ovariectomy. J. Vet. Med. Sci..

[12] Scott J., Singh A., Valverde A. (2020). Pneumoperitoneum in veterinary laparoscopy: A review. Vet. Sci..

[13] Umano G.R., Delehaye G., Noviello C., Papparella A. (2021). The “dark side” of pneumoperitoneum and laparoscopy. Minim. Invasive Surg..

[14] Park Y.T., Okano S. (2015). Influence of pneumoperitoneum and postural change on the cardiovascular and respiratory systems in dogs. J. Vet. Med. Sci..

[15] Fukushima F.B., Malm C., Andrade M.E.J., Oliveira H.P., Melo E.G., Caldeira F.M.C., Gheller V.A., Palhares M.S., Macedo S.P., Figueiredo M.S. (2011). Cardiorespiratory and blood gas alterations during laparoscopic surgery for intra-uterine artificial insemination in dogs. Can. Vet. J..

[16] Čechovičienė S., Šidlauskaitė I., Grigonis A., Karvelienė B., Sarapinienė I., Čiapienė I., Kerzienė S., Riškevičienė V., Juodžentė D. (2026). Effects of laparoscopic and open ovariectomy on cortisol and oxidative stress in dogs under the same anesthesia protocol. Vet. Sci..

[17] Lee J.Y., Choi S.H. (2015). Evaluation of total oxidant and antioxidant status in dogs under different CO_2_ pneumoperitoneum conditions. Acta Vet. Scand..

[18] Stańczyk M., Gromadzińska J., Wasowicz W. (2005). Roles of reactive oxygen species and selected antioxidants in regulation of cellular metabolism. Int. J. Occup. Med. Environ. Health.

[19] Lee Y., Song B.C., Yeum K. (2015). Impact of volatile anesthetics on oxidative stress and inflammation. BioMed Res. Int..

[20] Tomsič K., Nemec Svete A. (2022). A mini-review of the effects of inhalational and intravenous anesthetics on oxidative stress in dogs. Front. Vet. Sci..

[21] Mittal M., Siddiqui M.R., Tran K., Reddy S.P., Malik A.B. (2014). Reactive oxygen species in inflammation and tissue injury. Antioxid. Redox Signal..

[22] Dissemond J., Goos M., Wagner S.N. (2002). The role of oxidative stress in the pathogenesis and therapy of chronic wounds. Hautarzt.

[23] Azizi S., Kazemi Mehrjerdi H., Zaeemi M. (2025). The protective role of melatonin and agomelatine against oxidative stress following laparoscopic ovariectomy in dogs. BMC Vet. Res..

[24] Kim S.H., Park J. (2019). IDH2 deficiency impairs cutaneous wound healing via ROS-dependent apoptosis. Biochim. Biophys. Acta Mol. Basis Dis..

[25] Milech V., de Oliveira J.S., de Ataide M.A.W., Coradini G.P., Mann T.R., Sarturi V.Z., Hartmann H.F., Linhares M.T., Antunes B.N., de Andrade C.M. (2021). Effects of heated pneumoperitoneum on inflammation, oxidative stress, and peritoneal histology in female dogs that underwent video-assisted ovariohysterectomy. Vet. Med. Int..

[26] Costa G.L., Leonardi F., Licata P., Porcino M., De Paoli F., Iannelli D., Bruno F., Macrì F., Iannelli N.M. (2025). Ovariectomy in canine surgical medicine: A comparative analysis of surgical approaches and the nociceptive, inflammatory, and oxidative stress responses. Animals.

[27] Kumari A., Tiwary R., Guha S.K., Kumar R., Kumar R. (2022). Oxidative stress and antioxidant activity in female dogs undergoing laparoscopic and open elective ovariectomy. Indian J. Anim. Sci..

[28] Dalmolin F., Rubio C.P., Furlanetto C.S., Steffens R., Hadi N.I.I.A., da Silva A.d.L., Tomazi P., Antunes B.N., Elias F., Schmidt E.M.d.S. (2024). Changes in biomarkers of inflammation and oxidative status in dogs subjected to celiotomy or video-assisted ovariohysterectomy. Vet. Sci..

[29] Naghavi R., Kazemi Mehrjerdi H., Heidarpour M. (2025). Assessment of pain, vital parameters and oxidative stress markers in dogs after celiotomy and three-port laparoscopic ovariectomy. Vet. Med. Sci..

[30] Lee J.Y., Won H.S., Hwang H.K., Jeong S.M., Kim M.C. (2013). Evaluation of the systemic oxidative stress status during major orthopedic surgery in dogs: A clinical study. J. Vet. Clin..

[31] Calabria A., Del Prete C., Pasolini M.P., Palumbo V., Nappo M.A., Ammirati S., Ciccarelli D., Micieli F., Kosior M.A., Carbone C. (2026). Surgical impact of laparoscopic and laparotomic elective ovariectomy on inflammatory and oxidative stress biomarkers in anestrus dogs. Vet. Surg..

[32] Devitt C.M., Cox R.E., Hailey J.J. (2005). Duration, complications, stress, and pain of open ovariohysterectomy versus a simple method of laparoscopic-assisted ovariohysterectomy in dogs. J. Am. Vet. Med. Assoc..

[33] Hancock R.B., Lanz O.I., Waldron D.R., Duncan R.B., Broadstone R.V., Hendrix P.K. (2005). Comparison of postoperative pain after ovariohysterectomy by harmonic scalpel-assisted laparoscopy compared with median celiotomy and ligation in dogs. Vet. Surg..

[34] Alipour F., Emami M.R., Mohri M. (2018). Endocrine and oxidative stress characteristics in different anesthetic methods during pneumoperitoneum in dogs. Comp. Clin. Pathol..

[35] Prete A., Yan Q., Al-Tarrah K., Akturk H.K., Prokop L.J., Alahdab F., Foster M.A., Lord J.M., Karavitaki N., Wass J.A. (2018). The cortisol stress response induced by surgery: A systematic review and meta-analysis. Clin. Endocrinol..

[36] Kumar R., Bishnoi P., Tanwar M., Jhirwal S.K., Kumari A. (2023). Haemato-biochemical changes after laparoscopic and conventional ovariohysterectomy in dogs. Indian J. Vet. Sci. Biotechnol..

[37] Okur D.T., Polat B. (2021). Comparison of the postoperative outcome of the three-port laparoscopic ovariectomy and conventional open ovariectomy methods in dogs. Thai J. Vet. Med..

[38] Kohl B.A., Deutschman C.S. (2006). The inflammatory response to surgery and trauma. Curr. Opin. Crit. Care.

[39] Bain C.R., Myles P.S., Martin C., Wallace S., Shulman M.A., Corcoran T., Bellomo R., Peyton P., Story D.A., Leslie K. (2023). Postoperative systemic inflammation after major abdominal surgery: Patient-centred outcomes. Anaesthesia.

[40] Jiménez A.G., Strasser R. (2025). Effects of adverse life history on oxidative stress and cytokine concentration in domestic dogs. J. Appl. Anim. Welf. Sci..

[41] Rubio C.P., Saril A., Kocaturk M., Tanaka R., Koch J., Cerón J.J., Yilmaz Z. (2020). Changes of inflammatory and oxidative stress biomarkers in dogs with different stages of heart failure. BMC Vet. Res..

[42] Xie Y., Yang M., Huang J., Jiang Z. (2025). Identification and characterization of genes associated with intestinal ischemia-reperfusion injury and oxidative stress: A bioinformatics and experimental approach integrating high-throughput sequencing, machine learning, and validation. J. Inflamm. Res..

[43] Menger M.D., Vollmar B. (2004). Surgical trauma: Hyperinflammation versus immunosuppression?. Langenbecks Arch. Surg..

[44] Hernández-Avalos I., Mota-Rojas D., Miranda-Cortés A.E., Casas-Alvarado A., Flores-Gasca E., Domínguez-Oliva A. (2021). Neurobiology of anesthetic-surgical stress and induced behavioral changes in dogs and cats: A review. Vet. World.

[45] Mizuno T., Kamiyama H., Mizuno M., Mizukoshi T., Shinoda A., Harada K., Uchida S., Lee J.S., Kasuya A., Sawada T. (2015). Plasma cytokine levels in dogs undergoing cardiopulmonary bypass. Res. Vet. Sci..

[46] Avazi D., Awasum A. (2024). Investigation of the time-course of tumour necrosis factor-α in dogs with cutaneous wounds. J. Biol. Res. Rev..

[47] Yamashita K., Fujinaga T., Miyamoto T., Hagio M., Izumisawa Y., Kotani T. (1994). Canine acute phase response: Relationship between serum cytokine activity and acute phase protein in dogs. J. Vet. Med. Sci..

[48] Hasson H.M. (1999). Open laparoscopy as a method of access in laparoscopic surgery. Gynaecol. Endosc..

[49] Rubio C.P., Tvarijonaviciute A., Caldin M., Hernández-Ruiz J., Cerón J.J., Martínez-Subiela S., Tecles F. (2018). Stability of biomarkers of oxidative stress in canine serum. Res. Vet. Sci..

[50] Total Antioxidant Status (TAS) Colorimetric Assay Kit. https://documents.thermofisher.com/TFS-Assets/BID/manuals/EEA025%C2%A0K801-M-manual-metabolic-A5-2024.6.11.pdf.

[51] Total Oxidant Status (TOS) Colorimetric Assay Kit. https://documents.thermofisher.com/TFS-Assets/BID/manuals/EEA027%20K802-M-manual-metabolic-A5-2024.6.11.pdf.

[52] Baysal E., Taysi S., Aksoy N., Uyar M., Celenk F., Karatas Z.A., Tarakcioglu M., Bilir H., Mumbuc S., Kanlikama M. (2012). Serum paraoxonase, arylesterase activity and oxidative status in patients with obstructive sleep apnea syndrome (OSAS). Eur. Rev. Med. Pharmacol. Sci..

[53] Canine TNF-Alpha (TNF) ELISA. https://documents.thermofisher.com/TFS-Assets%2FLSG%2Fmanuals%2FECTNF.pdf.

[54] Canine IL-6 ELISA. https://documents.thermofisher.com/TFS-Assets%2FLSG%2Fmanuals%2FECIL6.pdf.

[55] Church D.B., Nicholson A.I., Ilkiw J.E., Emslie D.R. (1994). Effect of non-adrenal illness, anaesthesia and surgery on plasma cortisol concentrations in dogs. Res. Vet. Sci..

[56] Naddaf H., Najafzade Varzi H., Sabiza S., Falah H. (2014). Effects of xylazine-ketamine anesthesia on plasma levels of cortisol and vital signs during laparotomy in dogs. Open Vet. J..

[57] Restitutti F., Raekallio M., Vainionpää M., Kuusela E., Vainio O. (2012). Plasma glucose, insulin, free fatty acids, lactate and cortisol concentrations in dexmedetomidine-sedated dogs with or without MK-467: A peripheral α2-adrenoceptor antagonist. Vet. J..

[58] Li L., Dong J., Fen X., Li B., Chen Y., Sha J., Fan H. (2017). Effects of dexmedetomidine on plasma glucose, cortisol and adrenocorticotropic hormone concentrations in canines undergoing ovariohysterectomy. Thai J. Vet. Med..

[59] Hunt A., Olin S., Whittemore J.C., Esteller-Vico A., Springer C., Giori L. (2024). The effects of selected sedatives on basal and stimulated serum cortisol concentrations in healthy dogs. PeerJ.

[60] Salzman J., Olin S., Esteller-Vico A., Giori L. (2025). Duration of sedation effects on the ACTH stimulation test in healthy dogs. PLoS ONE.

[61] Rubio C.P., Hernández-Ruiz J., Martinez-Subiela S., Tvarijonaviciute A., Ceron J.J. (2016). Spectrophotometric assays for total antioxidant capacity (TAC) in dog serum: An update. BMC Vet. Res..

[62] Perez-Montero B., Fermin-Rodriguez M.L., Portero-Fuentes M., Sarquis J., Caceres S., Illera del Portal J.C., de Juan L., Miro G., Cruz-Lopez F. (2024). Serum total antioxidant status in dogs: Reference intervals and influence of multiple biological and analytical factors. Vet. Clin. Pathol..

[63] Szczubial M., Kankofer M., Bochniarz M., Dąbrowski R. (2015). Effects of ovariohysterectomy on oxidative stress markers in female dogs. Reprod. Domest. Anim..

[64] Thomas A., Karayannopoulou M., Anagnostou T., Psalla D., Ioannou K., Ginoudis A., Savvas I., Pardali D. (2025). Study of oxidant/antioxidant profile in dogs with mammary cancer undergoing mastectomy during the peri-operative period. Vet. Sci..

[65] Perez-Montero B., Fermin-Rodriguez M.L., Portero-Fuentes M., Sarquis J., Caceres S., Portal J.C., de Juan L., Miro G., Cruz-Lopez F. (2025). Malondialdehyde (MDA) and 8-hydroxy-2′-deoxyguanosine (8-OHdG) levels in canine serum: Establishing reference intervals and influencing factors. BMC Vet. Res..

[66] Blanca P.M., María Luisa F.R., Guadalupe M., Fátima C.L. (2024). Oxidative stress in canine diseases: A comprehensive review. Antioxidants.

[67] Del Prete C., Calabria A., Palumbo V., Nappo M.A., Ammirati S., Ciccarelli D., Micieli F., Kosior M.A., Carbone C., Cocchia N. (2025). Comparison of outcomes between laparotomic and laparoscopic elective ovariectomy in anestrus dogs: Postoperative recovery, pain and inflammatory biomarkers. Vet. Res. Commun..

[68] Avazi D.O., Awasum A.C., Hassan A.Z., Ayo J.O., Aluwong T., Muhammed S.T., Simon A.Y., Suleiman M.H., Kudi A.C. (2019). Evaluation of levels of interleukin-6, interleukin-8 and some haematologic parameters of dogs with cutaneous wounds. Cytokine.

[69] Nezu Y., Shigihara K., Harada Y., Yogo T., Hara Y., Tagawa M. (2008). Effects of small intestinal ischemia and reperfusion on expression of tumor necrosis factor-α and interleukin-6 messenger RNAs in the jejunum, liver, and lungs of dogs. Am. J. Vet. Res..

[70] Yuki K. (2021). The immunomodulatory mechanism of dexmedetomidine. Int. Immunopharmacol..

[71] Li B., Li Y., Tian S., Wang H., Wu H., Zhang A., Gao C. (2015). Anti-inflammatory effects of perioperative dexmedetomidine administered as an adjunct to general anesthesia: A meta-analysis. Sci. Rep..

[72] Merlin T., Cinti F., Charlesworth T.M. (2022). Healthy nonobese bitches maintain acceptable spontaneous ventilation during laparoscopic ovariectomies. J. Am. Vet. Med. Assoc..

[73] Fernández-Martín S., Valiño-Cultelli V., González-Cantalapiedra A. (2022). Laparoscopic versus open ovariectomy in bitches: Changes in cardiorespiratory values, blood parameters, and sevoflurane requirements associated with the surgical technique. Animals.

[74] Cardoso C.G., Marques D.R., da Silva T.H., de Mattos-Junior E. (2014). Cardiorespiratory, sedative and antinociceptive effects of dexmedetomidine alone or in combination with methadone, morphine or tramadol in dogs. Vet. Anaesth. Analg..

[75] Bustamante R., Canfrán S., Gómez de Segura I.A. (2024). Clinical evaluation of the sedative, antinociceptive and cardiorespiratory effects of intranasal dexmedetomidine combined with methadone in healthy dogs. Vet. J..

[76] Nishimura L.T., Auckburally A., Santilli J., Vieira B.H.B., Garcia D.O., Honsho C.S., de Mattos-Junior E. (2018). Effects of dexmedetomidine combined with commonly administered opioids on clinical variables in dogs. Am. J. Vet. Res..

